# Older Learners in Higher Education: Age-Friendly University Models in and Beyond the Classroom (A Collaborative Symposium between the AFU and Intergenerational Learning, Research and Community Engagement Interest Groups)

**DOI:** 10.1093/geroni/igaa057.1814

**Published:** 2020-12-16

**Authors:** Skye Leedahl

## Abstract

The Age-Friendly University (AFU) movement is specifically targeting one group of adult learners who are less represented within higher education -- individuals considered “older adults,” with five of the ten principles focused on promoting educational opportunities for older adult learners. However, there is less understanding within higher education for how to ensure inclusivity of this group. Importantly, some universities across the country have identified promising strategies for engaging older adult learners within higher education classrooms and supporting them beyond the classroom. As this intergenerational learning model continues to grow, there is much to learn from those who have begun efforts to appropriately utilize and engage older adult learners. This symposium will highlight examples from universities that have identified ways to create age-diverse programs within the university setting. The first paper will begin by discussing intergenerational learning opportunities for utilizing older adult learners in innovative ways to enhance university student experiences, and the second paper will specifically highlight successful activities used in a university class to engage older and younger adult learners. The third paper will examine ways in which a university and Osher Lifelong Learning Institute work together and promote research opportunities for both generations. The fourth paper will discuss research conducted to investigate how intergenerational classroom experiences are shaped by older adults. The fifth paper will describe the use of technology training workshops to promote service learning for university students and those in a retirement community. This would be a collaborative symposium between the AFU and ILRCE Interest Groups.

